# Environmental Influences on Physical Activity among Rural Adults in Montana, United States: Views from Built Environment Audits, Resident Focus Groups, and Key Informant Interviews

**DOI:** 10.3390/ijerph14101173

**Published:** 2017-10-04

**Authors:** Brian K. Lo, Emily H. Morgan, Sara C. Folta, Meredith L. Graham, Lynn C. Paul, Miriam E. Nelson, Nicolette V. Jew, Laurel F. Moffat, Rebecca A. Seguin

**Affiliations:** 1 Division of Nutritional Sciences, Cornell University, Ithaca, NY 14850, USA; bl592@cornell.edu (B.K.L); ehm72@cornell.edu (E.H.M.); mlg22@cornell.edu (M.L.G.); nvj4@cornell.edu (N.V.J.); lfm53@cornell.edu (L.F.M.); 2 Friedman School of Nutrition Science and Policy, Tufts University, Boston, MA 02111, USA; sara.folta@tufts.edu; 3 College of Education, Health and Human Development, Montana State University, Bozeman, MT 59717, USA; lpaul@montana.edu; 4 Sustainability Institute, University of New Hampshire, Durham, NH 03824, USA; miriam.nelson@unh.edu

**Keywords:** physical activity, rural health, triangulation, mixed methods, built environment, obesity, prevention

## Abstract

Rural populations in the United States have lower physical activity levels and are at a higher risk of being overweight and suffering from obesity than their urban counterparts. This paper aimed to understand the environmental factors that influence physical activity among rural adults in Montana. Eight built environment audits, 15 resident focus groups, and 24 key informant interviews were conducted between August and December 2014. Themes were triangulated and summarized into five categories of environmental factors: built, social, organizational, policy, and natural environments. Although the existence of active living features was documented by environmental audits, residents and key informants agreed that additional indoor recreation facilities and more well-maintained and conveniently located options were needed. Residents and key informants also agreed on the importance of age-specific, well-promoted, and structured physical activity programs, offered in socially supportive environments, as facilitators to physical activity. Key informants, however, noted that funding constraints and limited political will were barriers to developing these opportunities. Since building new recreational facilities and structures to support active transportation pose resource challenges, especially for rural communities, our results suggest that enhancing existing features, making small improvements, and involving stakeholders in the city planning process would be more fruitful to build momentum towards larger changes.

## 1. Introduction

More than half of Americans do not achieve the current recommended levels of physical activity [1], and rural populations are even less likely to meet the guidelines compared to their urban and peri-urban counterparts [2,3]. Geographic disparities in physical activity may be driven in part by environmental factors in rural communities that limit opportunities to be active, including poor quality, limited availability, and inadequate access to recreational facilities, as well as geographic and topographic features that inhibit active living and transportation [4,5].

In the past decade, there has been a proliferation of interest in understanding the relationship between built environments and physical activity [6,7,8], and identifying effective strategies to promote active living [9,10,11,12]. However, much of the evidence supporting policy and environmental strategies to encourage active living comes from research in non-rural settings [6,9,10,11]. Rural communities have several features relevant to the built environment that set them apart from more densely populated areas [13,14], including more dispersed populations, longer distances between destinations, lack of public transportation, distinct social norms and cultural practices, and different recreational environments. Further, rural communities have higher poverty rates and lower income levels than urban areas [15], which impacts individual opportunities, as well as tax revenues and funds available for improving active living structures. Given the unique physical and contextual challenges faced by rural communities, special considerations should be given to them when developing strategies to enhance physical activity participation in rural settings [14]. Existing policies and strategies to support active living through environmental changes, however, are mostly urban-oriented. For example, although national recommendations, such as the Common Community Measures for Obesity Prevention (COCOMO) published by the Centers for Disease Control and Prevention (CDC), are in place to guide the improvement of physical activity engagement, many of them are not applicable to rural communities [14]. These published strategies largely relate to proximity to schools, enhanced walking and biking infrastructure, improvement of public transportation, mixed-land use, and improved personal and traffic safety where people are usually physically active [16].

Physical activity is a multifactorial behavior and is influenced by a wide range of factors related to a person’s surroundings [17]. Objective aspects of the environment as well as individuals’ perceptions of their environment are likely to be important influences [17]. As rural communities are highly heterogeneous, factors influencing rural physical activity vary substantially across rural built environment studies, depending on the geographic and social contexts of the population being studied [8,18,19,20,21]. Given this, it is advantageous to use a variety of data sources to understand the various dimensions and support a more nuanced interpretation of barriers and facilitators. Focus groups with residents are an effective way to explore a variety of issues in a community that influence behavior [22], while interviews with key informants can be used to gather additional information and provide further insights [23]. Data from objective environmental audits can elucidate complementary or contradictory aspects of the issues identified from focus groups and key informants’ interviews [24,25].

Therefore, the purpose of the present study was to gather information from built environment audits, resident focus groups, and key informant interviews, to capture different factors that influence rural adults’ physical activity engagement. As building new active living infrastructures often poses resource challenges to rural communities, our hope is to identify strategies that could potentially leverage rural communities’ exiting assets and resources to improve rural health.

## 2. Materials and Methods

**Figure 1:**
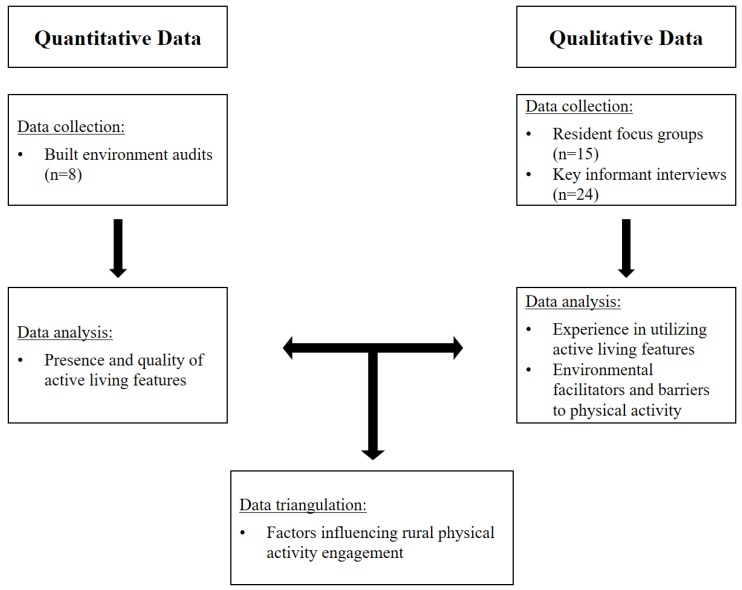
Data collection, analysis, and interpretation process.

Figure 1 shows the overall data collection and analysis process. Data were collected and analyzed between 2014 and 2017 as part of formative research for the Strong Hearts, Healthy Communities trial, a rural community-based cardiovascular disease prevention program [26] in eight government-designated medically underserved rural Montana towns [16]. The study was approved by the Cornell University Institutional Review Board (Protocol #1402004505).

### 2.1. Setting

Montana is one of the most rural states in the United States, with close to two thirds of the population living in rural areas (64.7%) [27]. As of 2013, only 23.3% of the state’s adult population met the national physical activity guidelines [28]. Study towns were selected to represent different geographic regions.

Table 1 shows the demographics of the study towns. In all towns, most people were non-Hispanic white and the median household income was below $50,000. Population density ranged from 213 to 1029 people per square kilometer, with a median age between 37.9 and 55.4 years [29]. To illustrate the rurality of our study towns, demographics of New York City are included in Table 1 for comparison.

### 2.2. Built Environment Audits

In each town, we used a reliable community asset inventory tool [31], Inventories for Community Health Assessment in Rural Towns (iCHART), to assess active living characteristics related to physical activity opportunities. The tool organizes built environment characteristics into 11 categories: the presence of retail business, professional services, community services, town amenities, physical activity facilities, town aesthetics, condition of sidewalks, condition of the town center, condition of street and intersections, street and intersection safety features, and biking facilities. As mixed land use and active living characteristics are associated with physical activity [8,11], the assessment of these characteristics allows the understanding of the strengths and weaknesses of the built environment of the study towns. We also assessed the presence of stray animals given previous research indicating their relevance to outdoor activity safety in the midwest United States [18]. The iCHART tool contains a checklist of items that the researcher looks for and documents during the audit. Table S1 shows the individual items assessed within each built environment category.

In all towns, Meredith L. Graham (MLG) and either a local National Institute of Food and Agriculture (NIFA) extension agent or another research team member conducted the audits on different days. The audits were completed in two steps: (1) a one-mile walking tour from town center and (2) a four-mile “windshield” tour. The windshield tour allowed for identification of built environment features that were difficult to observe on foot or may not be within walking distance. Discrepancies between audits were discussed for consensus.

### 2.3. Resident Focus Groups

NIFA extension agents and their local partners recruited overweight (body mass index (BMI) ≥ 25.0) and sedentary (<30 min of physical activity per week) adults aged ≥40 years to take part in focus group discussions. Recruitment strategies included press releases, flyers, website posts, word-of-mouth referrals, and direct contact with community residents. To confirm eligibility, we asked potential participants to complete a screening survey with questions about age, height, weight, and physical activity level.

Focus groups were stratified by age (40–64, 65+) and gender, as different responses were expected according to different age and gender groups. Discussions lasted between 60 and 90 min and were facilitated by MLG, an experienced qualitative researcher. The discussion guide was based on an ecological framework [32] (pp. 465–486) and developed by Meredith L. Graham (MLG), Sara C. Folta (SCF), and Rebecca A. Seguin (RAS) to explore participants’ attitudes, perceptions, barriers, and facilitators to physical activity. Prior to use, the discussion guide was pilot-tested and refined. Participants provided written informed consent and completed a brief demographic and health behavior questionnaire. Sessions were digitally recorded for transcription. Participants were compensated $50. Table 2 shows a sub-set of the focus group guide questions that were relevant to the present study.

### 2.4. Key Informant Interviews

In each town, we conducted phone interviews with three key informants (*n =* 24) identified by NIFA extension agents. Key informants represented diverse areas of community leadership, including recreation, local government, public health and healthcare, social services, community programming, and business. Because of the inherent confidentiality concerns in conducting research in small rural communities, key informant characteristics will not be reported in detail. The interview guide focused on locally relevant environmental influences on physical activity. As with the focus groups, we piloted and revised the interview guide prior to use. Most interviews lasted between 45 and 60 min and all were digitally recorded and transcribed verbatim. Key informants provided verbal consent and were compensated $25. Table 3 shows a sub-set of the interview guide questions that were relevant to the present study.

### 2.5. Analysis

We enumerated items identified through the built environment audits by category (Table S1).

Brian K. Lo (BKL) and Emily H. Morgan (EHM) performed thematic analysis of the qualitative data [33]. Guided by an ecological framework [32] (pp. 465–486), we developed an initial codebook using a “lumping” technique to look for overarching themes and coded transcripts into three broad categories of influences related to physical activity participation: individual, environmental, and socio-cultural influences [34]. Brian K. Lo (BKL) then used a “splitting” technique to look for more detailed themes or smaller categories within the three broader categories of influences [34]. A subsequent codebook relevant to the present study was then developed and it was continuously discussed and refined among the research team. Brian K. Lo (BKL) and a research assistant independently coded a subset of the data using the refined codebook with an observed agreement >90% between the coders. Any discrepancies were then discussed among the research team to reach consensus. Data were then recoded using the final codebook.

We first analyzed the focus group and interview data separately to identify major and minor themes in each dataset, and then compared and contrasted emerging themes. We organized themes into five categories of environmental factors guided by the Ecological Model of Active Living, developed by Sallis et al. [32] (pp. 465–486): built, social, organizational, policy, and natural environments. To facilitate data triangulation, we transformed results from the built environment audits into qualitative narrative summaries and then compared them with the themes identified in the focus group and interviews. We used NVivo (Mac 11, QSR International Pty Ltd., Doncaster, Victoria, Australia) to assist coding and analysis.

## 3. Results

A total of 118 adults, aged 40–91 years, participated in the focus group discussions. The socioeconomic characteristics of participants broadly aligned with the composition of the communities in which they lived. Although all participants were sedentary and outside of the optimal BMI range (mean BMI = 31.9 ± 5.77), three-fourths (75.4%) rated themselves having “good” to “excellent” health.

**Figure 2:**
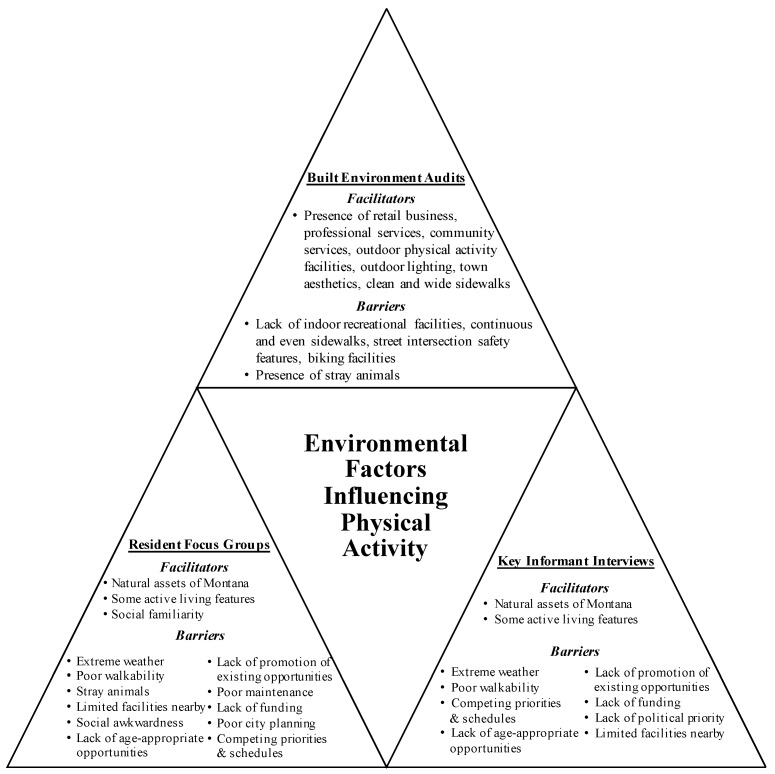
Sub-themes identified with each data source: built environment audits, resident focus groups, and key informant interviews.

Figure 2 below summarizes the sub-themes identified with each data source.

Built environment audits identified a range of active living assets in each town (Table S1). We identified retail businesses, professional services, community services, outdoor physical activity facilities, outdoor lighting, aesthetics, and clean and wide sidewalks in all towns. In many communities, we did not observe continuous and even sidewalks, street intersection safety features, or biking facilities. We documented stray animals in six out of eight of the towns. The presence of other built environment features, such as indoor recreational facilities and trails, varied considerably between communities.

### 3.1. Interpretation of Built Environment Audits, Focus Groups, and Interview Data

Emergent themes within the five categories of environmental influences were described in relation to the observed built environment characteristics. Representative quotes are presented in Table 4.

#### 3.1.1. Built Environment

In all focus groups, participants described the presence of physical activity facilities, such as sports fields, recreation centers, swimming pools, or gyms, confirming the findings of the built environment audits. Focus group respondents in several communities also described the common use of non-traditional or mixed-use spaces for physical activity, such as school athletic facilities and hotel swimming pools, although personal connections with facility management were sometimes required. For those who lived outside of town, distance hindered facility use. Focus group participants and key informants commonly expressed a desire for larger and more diverse recreational centers with a broader range of physical activity opportunities, especially in the winter.

Many participants specified that walking was the preferred form of physical activity. Participants’ comments reflected findings of the built environment audits that variation existed in the presence, condition, and safety of the sidewalks and streets in town centers. Several focus group and interview participants felt that investment was needed in pedestrian-friendly features, and that the lack of sidewalks, poor sidewalk quality, and the presence of stray animals were major barriers to walking outside. In addition, town walkability was subject to weather conditions, especially in communities where sidewalks were not adequately cleared of snow. 

#### 3.1.2. Social Environment

The audit tool did not capture social environments, but the focus groups identified them as crucial to rural residents’ usage of the available physical activity resources. The social environments at recreational facilities influenced some residents’ physical activity behaviors. Some participants perceived sports fields and gyms as youth spaces, and felt out of place in these spaces.

Several participants described a desire for fitness opportunities with people of a similar age and fitness levels. Some said that they felt uncomfortable exercising with younger and more physically fit people, while others referenced the camaraderie of a peer atmosphere. The desire for age-appropriate opportunities was supported by key informants’ complaints about the lack of adult-specific programs and activities in rural communities. The benefits of structured exercise classes with peers was discussed more commonly in focus groups with women than in groups with men. To some women, a socially familiar environment provided a sense of safety for physical activity.

#### 3.1.3. Organizational Environment

Facility and program schedules strongly influenced use and engagement. People who worked business hours or lived far from facilities reported that operating schedules often did not meet their needs. When multiple users shared facilities, access for children and youth was viewed as the priority.

To some male residents, the quality of facilities was an important factor, and outdated equipment was cited as a barrier to use. Further, both residents and key informants agreed that cost (e.g., membership fees, class fees) was a barrier to participating in some activities. For example, several participants reported that they enjoyed skiing, but found regional ski resorts to be cost-prohibitive.

In addition, information dissemination influenced participation in scheduled physical activity. Many participants described social networks—friends and family—as the means by which coordinated opportunities, such as pick-up basketball games and fitness classes, were promoted most often. Some described feeling excluded because they were less connected to networks in which this information would be shared. In addition, although many residents were able to identify local programs and opportunities, they were not always aware of specific schedules or content. Key informants suggested that the greater promotion of existing opportunities for physical activity would be helpful.

#### 3.1.4. Policy Environment

Residents cited poor city planning and lack of maintenance of existing facilities as barriers to physical activity. These comments often were related to perceptions that local governments do not have funding or interest in promoting physical activity to adults. In some towns, key informants also expressed this belief. However, although several key informants criticized the lack of sidewalks and sidewalk quality, in one community, the key informants reported improvements in these features, suggesting differences in political agenda and priorities between jurisdictions.

#### 3.1.5. Natural Environment

Although seasonal factors and the natural geography were not captured in the built environment audits, the natural geography of Montana, including its diverse terrain, open spaces, and water features, was raised in all focus groups, and in some interviews as an important facilitator for leisure-time physical activity, including hiking, running, skiing, hunting, and fishing. However, while the warmer months favored outdoor activities, extreme winter weather was described by focus group participants and key informants as a significant barrier to outdoor physical activity.

## 4. Discussion

The aim of the present study was to use information gathered from built environment audits, resident focus groups, and key informant interviews to understand factors that influence physical activity among sedentary, overweight, midlife and older adults living in rural communities. While built environment audits provided a blueprint of the characteristics of the built environment, resident focus groups and key informant interviews provided critical contextual information on how the rural built environment encourages or discourages physical activity.

In the eight towns sampled in this study, spaces for physical activity were available but were not always perceived to be “activity-friendly” for adults. For example, although some residents expressed their desire to use the athletics facilities at schools, these spaces are usually prioritized for use by youth sports. Competition for physical activity spaces in rural settings has not been discussed extensively in the literature, but suggests a need for shared- or open-use policies with schools outside school hours to enable structured physical activity programs that are tailored to adults [14,35,36]. In the focus groups and interviews, we commonly heard about the use of “non-traditional” spaces for physical activity. Through community audits, we identified a range of other possible spaces that potentially could be utilized for hosting physical activity programs, such as churches, libraries, and municipal buildings. Other studies have found that non-traditional facilities, such as community centers, churches, and worksites, often are used for both planned and spontaneous physical activities in rural communities [19,37]. As funding for new construction and facility management is limited, attention towards optimal utilization of existing facilities is warranted.

Although previous studies have found social support, such as accountability to family and friends, company from pets, and peer influences, to facilitate engagement in physical activity among rural adults [18,38], triangulation of our findings adds to the literature that social networks also play a critical role in promoting physical activity opportunities among rural residents. Events, classes, and activity groups often are organized informally and publicized by word-of-mouth and social media. Participants also described that local connections and promotions for access to physical activity equipment at private facilities, such as those in hotels and hospitals. Finding ways to broaden promotion of existing and emerging opportunities and to support the operators of private physical activity spaces in expanding access should be considered.

Previous research has found the aesthetics of rural towns to be associated with physical activity levels [39,40]. All the rural towns in this study had vibrant town centers with pedestrian-friendly features and facilities for physical activity. Nonetheless, poor city planning and inadequate maintenance of facilities were perceived to be barriers for using existing indoor and outdoor spaces. For people living outside of town, geographic dispersion and a lack of transportation hindered utilization of available assets in town centers. Instead, many favored outdoor activities in the countryside, such as hiking, skiing, hunting, and fishing. These findings suggest that land-use policies affecting spaces both within and outside of towns are critical for the promotion of physical activity. Active and early engagement of residents in local planning and management processes may help improve coherence between resident demands and policy decisions [41].

This study also identified several important considerations for future built environment research in rural communities. First, we found the qualitative research extended the findings from the built environment audits and enriched our understanding of how communities perceive and interact with existing built environment features. As rural physical activity is complex and multifactorial, using a single assessment approach may limit the breadth and application of findings.

Second, our findings suggest that some constructs in the built environment audit tool are not relevant to all rural towns. For example, some street and intersection safety features, and biking facilities were not observed, and were not mentioned in either the focus groups or the interviews. This differed from what has been identified in other rural studies [42,43], and could be attributable to the composition of our participant sample. Our findings also suggest a benefit to collecting more details about recreational facilities and their operational characteristics (e.g., distance to residents’ homes, hours of operation, and quality of equipment), because of the important role of these factors in facility utilization [35,37]. Additionally, in locations where outdoor activity is popular, it may be helpful to gather data on natural geography features that provide opportunities for exercise such as trails, lakes, rivers, and mountains.

Third, there may be a benefit to adapting rural built environment audit tools to capture more places where residents are physically active. For example, we observed good to excellent condition of sidewalks in all town centers, but learned from local residents that sidewalk maintenance and availability was often suboptimal in certain areas of town where they felt it was needed. It is possible that the community audits could be enhanced by separately assessing features in town centers and outside of town (rather than combining the results of the walking and windshield tours) or by consulting local residents about the places and spaces that they go before creating the community maps.

The present study has few limitations. First, data collection was limited to rural Montana. However, we believe that our purposeful selection of diverse towns likely improved the relevance of the findings to rural communities in regionally proximate states, such as Idaho, North Dakota, South Dakota, and Wyoming, where population characteristics are similar. Second, our research focused on mid-life and older sedentary adults. It is likely that younger and more active residents may have different perceptions of physical activity opportunities and may interact differently with the environment. Third, as our study was cross-sectional, it is possible that some seasonal factors may have been missed. Finally, because we did not specify the full range of types of physical activity (e.g., recreational, functional, leisure, etc.) that we wanted to learn about to study participants, they may have limited their discussions to the types of activity that are most often discussed in the popular press.

## 5. Conclusions

Our findings suggest that rural communities have a number of built environment assets that promote active lifestyles but that their potential may not be fully realized. Given resource constraints and competing priorities, building new recreational facilities and structures to support active transportation is unlikely in many rural communities. However, enhancing existing features (e.g., current facilities and natural assets) and identifying opportunities to maximize their use, such as increasing the promotion of classes and available spaces, and revisiting scheduling, could support physical activity and help build momentum towards larger changes. Involving residents along with other stakeholders in the city planning process should be a priority. Our experience suggests that future rural active living research would benefit from use a triangulation approach to enhance understanding of unique characteristics of rural communities and identify relevant strategies for improving physical activity opportunities.

## Figures and Tables

**Table 1:** Study town demographics.

Site	Population Size ^1^	Rural-Urban Commuting Area (RUCA) Codes ^2^	Population Density (Per Square Kilometer) ^1^	Median Age ^1^	Median Household Income ($) ^1^	% White ^1^	% Population Below Poverty Level ^1^
New York City	8,426,743	1.00	10,680	35.8	53,373	43.3	20.6
Town A	8796	7.00	1029	37.9	46,935	95.1	13.0
Town B	488	10.00	561	51.3	44,583	92.6	9.0
Town C	1313	10.00	296	53.3	30,595	67.6	20.6
Town D	3376	7.00	213	40.0	44,119	85.0	16.4
Town E	1696	10.00	362	48.1	38,529	93.5	13.3
Town F	1923	10.00	642	48.3	46,053	89.4	10.5
Town G	979	10.00	661	55.4	29,813	87.5	16.4
Town H	7302	7.00	473	43.1	40,619	93.5	12.7

^1^ Based on 2015 census data [29]; ^2^ Based on 2010 RUCA data [30]: code of 7 or higher is designated as a small town not adjacent to a metro area or a rural area.

**Table 2:** Focus group guide questions related to physical activity.

Topic	Questions
Community understanding	Tell me about programs and physical and social aspects of your community that promote physical activity.
Attitudes & Perceptions	Would you consider your community healthy? Tell me about that. Tell me about the types of physical activity that you do. Describe the intensity of the type of exercise that you do. What makes you choose the type of exercise that you do?
Barriers	Tell me about things that make physical activity more difficult. What would prevent you from getting more exercise?
Facilitators	Tell me about the things that would help make exercising become habit. What would help you to get more exercise?

**Table 3:** Interview guide questions related to physical activity.

Topic	Questions
Community understanding	What do you see as the biggest overall problems or issues facing your community currently?
Attitudes & Perceptions	Would you consider your community healthy? Tell me about that. What do you like most about your community? What are your least favorite aspects of your community?
Barriers	Tell me about policies, physical or social aspects in this community that make physical activity more difficult.
Facilitators	Tell me about programs, policies, physical and social aspects in this community that promote physical activity.
Programming	In your opinion, what could be done to improve the environment that would make it easier for people to be active? What types of opportunities or programs to improve their health might people in this community be interested in?

**Table 4:** Built, social, organizational, policy, and natural environment influences on physical activity engagement.

Environmental Influences	Representative Quotes
Built Environment	Facilitators:
	“Well, there are walking trails. They have a new trail downtown and up in the mule pasture. There’s (a) trail up there. And of course there‘re trails out in the forest all over.” (Town C, Men 40–64)
	“We do have some nice parks with parks, meaning jungle gyms and swing sets.” (Town H, Key Informant #1: Healthcare Provider)
	Barriers:
	“If we could come together as a community and try and build some sort of recreation center that would be open year round and that would be free to get into, or very minimal cost…I think that would really be helpful.” (Town H, Key Informant #1: Healthcare Provider)
	Town H participant A: “It was mentioned the other day at the Senior Center how dangerous the sidewalks and streets are around here.”
	Town H participant B: “Yeah, and on top of that, (Town H) never used to plow their roads…”
	(Town H, Women 65+)
Social Environment	Facilitators:
	“For me, myself, I find that I am more apt to exercise if I am in a group or have an organized program rather than saying I’m gonna do it on my own.” (Town B, Women 65+)
	“I think a lot of people are interested in doing [a group program] because they’re exercising with their peers, they’re not exercising at the local gym where there could be any age. I think these guys are all older and they really tailor to the older crowd and it’s all their buddies and somebody knows somebody in there on the treadmill or on the bike or whatever and I think if they had something like that available people would be more willing [to exercise].” (Town H, Key Informant #1: Healthcare Provider)
	Barriers:
	Town G participant A: “They opened up the gym, but it’s more of a young kids thing.”
	Town G participant B: “The adults go in there, you kinda feel outta place.”
	(Town G, Men 40–64)
	“We’re lacking some opportunities for adults to gather together and to participate in sporting endeavors.” (Town D, Key Informant #2: Political Leadership)
Organizational Environment	Facilitators:
	“If we had the facility to have a health club or whatever, where you could go when it was convenient for you, not when it was convenient for somebody else. And you could set your time and do it.” (Town F, Women 65+)
	“The school has an activity center and it’s free to community members. You just have to get a password and…you can go in and they have weight lifting and elliptical machines and treadmills.” (Town E, Key Informant #3: Local Leadership)
	Barriers:
	“You know, what the access is like… In our schools because I think they’re really busy, like gym and auditorium time is taken up with [school] sports.” (Town A, Key Informant #3: Healthcare Provider)
	“The weight gym…is a really a poor place. You know, there’s no TVs. You know, little cheap stereo like this, and the equipment’s older than me.” (Town C, Men 40–64)
Policy Environment	Facilitators:
	“The city has done some revamping of the streets…when they had redone the curb, they didn’t put in accessible curbs and, but now more recently they have done some other streets and they have done it and actually they’re very nice and very well done. And so, as far as accessibility, that has also improved. So I guess that is, yeah, that’s a good thing.” (Town A, Key Informant #3: Political Leadership)
	Barriers:
	“[Improving sidewalks] has been a concern of mine for years and I know it’s on the front of our city, but…It’s all about money.” (Town E, Key Informant #2: Educator)
	“I’m sure there are lots of ways [to make physical activity easier], but, I mean, I think the city could promote more public park space.” (Town H, Key Informant #3: Community Health Provider)
Natural Environment	Facilitators:
	“Because of the [natural] resources, we have available—downhill skiing, cross country skiing, snowshoeing, hunting, camping, hiking, fishing—you know, there’s a lot of opportunity for people to be active in this community and this area.” (Town H, Key Informant #2: Community-based Organization)
	“We’ve had a lotta people that’ve come in for, obviously, the trails and the hiking and the fishing and the skiing in the winter and the snowmobiling.” (Town H, Men 40–64)
	Barriers:
	“It’s hard to go for a walk when the wind’s blowin’ 50 miles an hour.” (Town G, Men 40–64)
	“Being where we’re at, weather can be a challenge. So, you may have weather during some of the months that may be warm enough, but with the winds that we get in this rural area…with the wind chill and just the wind, period, makes it difficult.” (Town D, Key Informant #3: Community Health Provider)

## Supplementary Materials

The following is available online at www.mdpi.com/1660-4601/14/10/1173/s1, Table S1: Individual item availability for the built environment audits.
