# Supplementary material for: Environmental Influences on Physical Activity among Rural Adults in Montana, United States: Views from Built Environment Audits, Resident Focus Groups, and Key Informant Interviews
Source: Int J Environ Res Public Health. 2017 Oct 4;14(10):1173. doi: 10.3390/ijerph14101173 (PMC5664674; doi:10.3390/ijerph14101173)
Supplement: Supplementary file 1 [file ijerph-14-01173-s001.pdf]

Supplementary materials

Table S1. Individual item availability for the built environment audits

Item	Town A	Town B	Town C	Town D	Town E	Town F	Town G	Town H
**Retail Business**								
Agricultural store	1 *	1	1	1	1	1	1	0 **
Appliance store	1	0	0	0	0	1	0	1
Automobile sales	1	1	1	1	1	1	1	1
Convenience store	1	1	1	1	1	1	1	1
Pharmacy	1	0	1	1	1	1	1	1
Florist	1	0	1	1	1	1	0	1
Furniture store	1	0	0	1	0	1	0	1
Gas station	1	1	1	1	1	1	1	1
Hardware store	1	1	1	1	1	1	1	1
Pawn shop	1	0	0	0	0	0	0	1
Retail store	1	1	1	1	1	1	1	1
Hunting store	1	1	0	0	1	0	0	1
Multi-service stores	1	1	1	1	1	1	1	1
**Professional services**								
Bank	1	0	1	1	1	1	1	1
Barber shop	1	1	1	1	1	1	1	1
Dry cleaning	0	0	0	0	0	0	0	1
Hotel	1	1	1	1	1	1	1	1
Laundromat	1	1	1	0	0	1	0	0
Post office	1	0	0	1	1	1	1	1
Train/bus station	0	0	0	1	0	1	0	1
**Community services**								
Church	1	1	1	1	1	1	1	1
Criminal justice	1	1	1	0	1	1	1	0
Day care	0	0	0	1	1	0	0	0
Entertainment	1	0	1	1	1	1	1	1
Health clinic	1	1	1	1	1	1	1	1
Library	1	1	1	1	1	1	1	1
Museum	0	1	1	0	0	0	1	1
Police station	1	1	1	1	1	0	1	0
School	1	1	1	1	1	1	1	1
Social service	1	1	1	1	1	1	1	1
Municipal building	1	1	1	1	1	1	1	1
**Town amenities**								
Benches	1	1	1	1	1	1	1	1
Trash cans	1	1	1	1	1	1	1	1
Water fountains	0	0	0	0	0	0	0	0
Covered bus stops	0	0	0	0	0	0	0	0
Outdoor lighting	1	1	1	1	1	1	1	1
**Physical activity facilities**								

Parks	1	1	1	1	1	1	1	1
Indoor recreational facilities	1	0	0	0	1	1	0	1
Playgrounds	1	1	1	1	1	1	1	1
Sports fields/courts	1	1	1	1	1	1	1	1
Pools	1	1	1	0	1	1	0	1
Trails	0	1	1	0	0	0	1	1
**Town aesthetics**								
Trees	1	1	1	1	1	1	1	1
Flowers	1	1	1	1	1	1	1	1
Decorative banners	1	1	1	1	0	0	0	1
Sculptures	0	1	0	0	0	0	0	1
Pleasant buildings	1	1	1	1	1	1	1	1
Awnings	1	1	1	0	1	1	0	1
Open space	1	1	1	1	1	0	0	1
**Sidewalk conditions**								
Sidewalk present	1	1	1	1	1	1	1	1
Sidewalk continuous	0	0	0	0	0	0	0	1
Sidewalk without slope	1	1	1	0	1	1	1	1
Sidewalk even	0	1	0	0	0	0	0	0
Sidewalk without obstructions	1	1	1	0	1	0	1	1
Sidewalk wide	1	1	1	1	1	1	1	1
Sidewalk clean	1	1	1	1	1	1	1	1
Sidewalk without graffiti	1	1	1	1	1	1	1	1
Sidewalk with connection	1	0	1	1	0	0	0	1
**Condition of town center**								
Litter	0	0	0	1	0	1	0	0
Graffiti	0	0	0	0	0	0	0	0
Unkempt lawns	1	0	0	1	0	0	1	0
Window bars	0	0	1	0	0	0	1	0
Broken windows	0	0	0	0	0	0	1	0
Vacant buildings	1	1	1	1	1	1	1	1
Vacant lots	1	1	1	1	1	1	1	1
Abandoned vehicles	0	1	1	0	1	1	1	0
Road kill	0	0	0	0	0	0	0	0
Dumpsters	1	1	0	1	1	1	1	1
Power plant	0	0	0	0	0	0	1	0
Landfill	0	0	0	0	0	0	0	0
**Street & intersection condition**								
Severe grades	0	0	0	0	0	0	0	0
Frequent curves	0	0	0	0	0	0	0	0
Numerous driveways	0	1	1	1	1	1	1	1
Narrow streets	0	0	0	0	0	0	1	0

Dumpsters streets	1	1	0	1	1	1	1	0
Sewers	0	0	0	0	0	0	0	0
**Street & intersection safety features**								
Stop light	1	0	0	0	0	1	0	1
In-road warning lights	0	0	0	0	0	0	0	0
Stop /yield sign	1	1	1	1	1	1	1	1
Posted speed limit	1	1	0	1	0	1	1	1
Pedestrian island	0	0	0	0	0	0	0	0
Crosswalk	1	1	1	1	1	1	1	1
Pedestrian crosswalk sign	1	0	0	0	0	0	0	1
In-road warning lights safety	0	0	0	1	0	0	0	0
Roundabouts	0	0	0	0	1	0	0	0
Curb bulb-outs	0	0	0	0	0	0	0	0
Speed-bumps brick roads	0	0	0	0	0	0	0	0
Pedestrian overpass/underpass	0	0	0	0	0	0	0	0
Midblock crosswalk	0	0	0	0	0	0	0	0
Raised crosswalk	0	0	0	0	0	0	0	0
Lane narrowing	0	0	0	0	0	0	0	0
Colored curb cuts	1	0	1	0	1	1	1	1
Tactile curb cuts	1	0	0	0	1	0	0	1
Emergency phone	0	0	0	0	0	0	0	0
Street lighting	1	1	1	1	1	1	1	1
Police	0	0	0	0	0	0	1	1
**Bicycle suitability**								
Bike lane	0	0	0	1	0	0	0	0
Bike racks	1	0	0	0	1	0	0	1
**Stray animals**								
Dangerous animals	1	1	1	0	0	1	1	1

* 1 indicates the item was observed as present.

** 0 indicates the item was not observed as present.
